# Hyperoxic Brain Effects Are Normalized by Addition of CO_2_

**DOI:** 10.1371/journal.pmed.0040173

**Published:** 2007-05-22

**Authors:** Paul M Macey, Mary A Woo, Ronald M Harper

**Affiliations:** 1 Department of Neurobiology, David Geffen School of Medicine, University of California Los Angeles, Los Angeles, California, United States of America; 2 School of Nursing, University of California Los Angeles, Los Angeles, California, United States of America; University College London, United Kingdom

## Abstract

**Background:**

Hyperoxic ventilation (>21% O_2_) is widely used in medical practice for resuscitation, stroke intervention, and chronic supplementation. However, despite the objective of improving tissue oxygen delivery, hyperoxic ventilation can accentuate ischemia and impair that outcome. Hyperoxia results in, paradoxically, increased ventilation, which leads to hypocapnia, diminishing cerebral blood flow and hindering oxygen delivery. Hyperoxic delivery induces other systemic changes, including increased plasma insulin and glucagon levels and reduced myocardial contractility and relaxation, which may derive partially from neurally mediated hormonal and sympathetic outflow. Several cortical, limbic, and cerebellar brain areas regulate these autonomic processes. The aim of this study was to assess recruitment of these regions in response to hyperoxia and to determine whether any response would be countered by addition of CO_2_ to the hyperoxic gas mixture.

**Methods and Findings:**

We studied 14 children (mean age 11 y, range 8–15 y). We found, using functional magnetic resonance imaging, that 2 min of hyperoxic ventilation (100% O_2_) following a room air baseline elicited pronounced responses in autonomic and hormonal control areas, including the hypothalamus, insula, and hippocampus, throughout the challenge. The addition of 5% CO_2_ to 95% O_2_ abolished responses in the hypothalamus and lingual gyrus, substantially reduced insular, hippocampal, thalamic, and cerebellar patterns in the first 48 s, and abolished signals in those sites thereafter. Only the dorsal midbrain responded to hypercapnia, but not hyperoxia.

**Conclusions:**

In this group of children, hyperoxic ventilation led to responses in brain areas that modify hypothalamus-mediated sympathetic and hormonal outflow; these responses were diminished by addition of CO_2_ to the gas mixture. This study in healthy children suggests that supplementing hyperoxic administration with CO_2_ may mitigate central and peripheral consequences of hyperoxia.

## Abstract 2

Hyperoxic ventilation leads to responses in brain areas that modify hypothalamus-mediated sympathetic and hormonal outflow; these responses can be diminished by addition of CO_2_ to the gas mixture.

## Editors' Summary

**Background.:**

All cells in the human body need oxygen (O_2_) to keep them alive. O_2_ is absorbed into the blood from the air by the lungs (which also release carbon dioxide [CO_2_], a waste product of cells, from the blood into the air). The blood then delivers O_2_ to the rest of the body. For healthy people, breathing air (which contains 21% O_2_) is sufficient to keep their tissues healthy. But there are medical situations in which O_2_ delivery to tissues needs improving. For example, during resuscitation or after a stroke when the O_2_ supply to a part of the brain is disrupted. Premature babies often need help with O_2_ delivery because their immature lungs don't absorb O_2_ efficiently. In situations like these, the O_2_ supply can be increased by providing an O_2_-rich gas mixture to the lungs—so-called “hyperoxic (i.e., high O_2_) ventilation.” But, paradoxically, hyperoxic ventilation can make matters worse. Hyperoxia increases the exchange of air between the lungs and the atmosphere (hyperventilation), which reduces the CO_2_ level in the blood. This “hypocapnia,” i.e. low CO_2_, reduces the blood flow to the brain by narrowing the blood vessels. Hyperoxia also alters the heart rate and blood pressure and the blood levels of some hormones. It probably causes these changes by affecting the brain regions that control autonomic functions (body functions such as heart rate, insulin and other hormone release, sweating and gland action that are not controlled by conscious thought). All told, although hyperoxic ventilation saves lives, it can also have serious adverse effects. In premature babies, for example, although it is often essential for their survival, hyperoxic ventilation can cause serious heart muscle and brain injury or lung problems (bronchopulmonary dysplasia) if it is not carefully monitored.

**Why Was This Study Done?:**

The addition of a little CO_2_ to the hyperoxic gas mix can reduce the adverse effects of hyperoxic ventilation on blood flow to the brain. However, it is unclear whether this alteration can also modify responses of brain areas that control autonomic functions and hormone release to hyperoxia. If it does, then CO_2_ supplementation could prevent those adverse effects of hyperoxic ventilation that affect the whole body. In this study, the researchers investigated whether hyperoxic ventilation increases neural responses in brain regions that regulate the activity of the hypothalamus (the part of the brain that controls autonomic bodily functions) and whether the addition of CO_2_ reduces these responses.

**What Did the Researchers Do and Find?:**

The researchers used a technique called functional magnetic resonance imaging (fMRI) to measure the activity of different brain regions in 14 healthy young people (aged 8–15 years). Active regions of the brain draw more O_2_ out of the blood than inactive regions, and fMRI measures changes in blood O_2_ levels. fMRI images were obtained for all the study participants when they were breathing normal air and during two-minute challenges with 100% O_2_ or a 95% O_2_, 5% CO_2_ mix. Hyperoxic ventilation produced rapid and marked changes in the activity of brain areas involved in autonomic and hormonal control, including the hypothalamus and regions that control the hypothalamus. After the challenge with 95% O_2_, 5% CO_2_, these responses were either absent or greatly reduced in the brain regions that had responded to 100% O_2_.

**What Do These Findings Mean?:**

These findings show that hyperoxic ventilation induces brain activity changes that are likely to affect autonomic functions and hormone release throughout the body. In addition, they show that the addition of CO_2_ to the gas mixture greatly diminishes these responses. Because the autonomic and hormonal changes induced by 100% O_2_ can potentially injure organs throughout the body, the addition of CO_2_ to hyperoxic gas mixtures could reduce many of the adverse effects of hyperoxic ventilation. These results, therefore, could influence how hyperoxic ventilation is used in medical practice. However, CO_2_ supplementation still needs to be tested in adults and newborn babies. Although the results presented here will probably hold true for adults, and both neonatal and developmental animal studies suggest that hyperoxia results in serious side effects in newborns over room air or hyperoxia with added CO_2_, the brain findings need to studied in babies, the portion of the population most likely to be treated with hyperoxic ventilation.

**Additional Information.:**

Please access these Web sites via the online version of this summary at http://dx.doi.org/10.1371/journal.pmed.0040173

- The American Lung Association has patient information on the lungs and lung diseases, including bronchopulmonary dysplasia (in English and Spanish)
- The Medlineplus encyclopedia contains pages on hyperventilation and on premature babies, and links to other information on premature babies (in English and Spanish)
- Wikipedia has pages on the lungs, oxygen toxicity, mechanical ventilation, and hypocapnia (note that Wikipedia is a free online encyclopedia that anyone can edit; available in several languages)

## Introduction

Hyperoxic ventilation (>21% O_2_) is widely used in medical practice, both in acute applications, such as resuscitation and stroke, and for chronic ventilatory supplementation. However, despite the objective of improving tissue oxygen delivery, hyperoxia can accentuate ischemia and impair that outcome. After initially suppressing ventilation through peripheral chemoreceptor action, hyperoxic gas mixtures paradoxically increase ventilation [1], likely by exciting chemosensitive brainstem neurons, possibly by an O_2_ free radical mechanism [2]. Reduced CO_2_–hemoglobin binding from increased O_2_ (the Haldane effect [3]) may additionally contribute to enhanced ventilation. The increase in ventilation due to hyperoxic exposure occurs also in neonates [4] and is long-lasting [5]. The hyperoxia-induced ventilatory increases result in reduced P_CO2_ and diminished cerebral blood flow (CBF) [1]. Oxygen delivery is further hindered by increasing the affinity of hemoglobin for O_2_ and reducing O_2_ unloading to tissues [6].

Hyperoxic challenges elicit local effects on brainstem sites, but also modify neural regulatory areas that mediate autonomic outflow and hormonal release. Significant increases in plasma glucagon and insulin levels and reduced myocardial contractility and relaxation result from even short hyperoxia exposures [7], and modifications in heart rate and blood pressure with hyperoxia are common [8,9]. Such hormonal and cardiovascular effects require involvement of central autonomic and hormonal regulation areas.

The particular brain structures recruited to influence hypothalamic and other autonomic and hormonal outflow responses to hyperoxia have been outlined by a large body of neuroanatomic and electrophysiologic data in animal models and functional magnetic resonance imaging (fMRI) studies of cold pressor or ventilatory challenges in humans [10–15]. Hypothalamic contributions to hormonal and sympathetic nervous system outflow are not regulated in isolation, but depend on multiple limbic and cortical regions, including insular, cingulate cortex, and hippocampal projections to particular hypothalamic sites [16,17]. The resulting hypothalamic influences on sympathetic ventral medullary final common path regions are modulated by such structures as the cerebellar cortex and deep nuclei, which limit or dampen extreme sympathetic changes (e.g., those occurring during profound decreases in blood pressure), and the insular cortex, which exerts lateralized influences on baroreceptor reflexes and other autonomic patterns [18]. Several limbic and cortical sites, including the insula and cingulate cortex, contribute to modulating the hypothalamo-pituitary-adrenocortical response to stress [19].

CBF reduction due to hyperoxia can be mitigated by adding CO_2_ to the gas mixture [6]. Elevations in CO_2_ lead to vasodilation of cerebral arteries and increased blood flow [20], and the addition of CO_2_ can alleviate a principal consequence of hyperoxic exposure—hypocapnia [6]. It is unclear, however, whether addition of CO_2_ modifies responses of brain areas that regulate sympathetic and hormonal outflow, potentially altering the central response of hyperoxia on peripheral vascular and organ responses, or whether the CO_2_ effects are confined to cerebral vasodilation, with little consequence to essential neural responses to hyperoxia. We hypothesized that hyperoxic exposure would elicit elevated responses in brain areas that regulate hypothalamic action, and that the magnitude of fMRI signals in brain areas mediating sympathetic and hormonal control should be diminished with the addition of CO_2_. Although it would be desirable to evaluate these issues in neonates, who are most likely to be exposed to resuscitation with hyperoxia, logistic issues with movement control in an environment not appropriate for sedation precluded use of such participants. We thus used an older group of children to investigate our hypotheses.

## Methods

### Participants and Informed Consent

Participants were 14 healthy children (age in years: mean 11, standard devation 2, range 8–15; seven male, seven female) participated. No participant had any diagnosis or evidence of respiratory or other disorders. Participants and parents or guardians provided written consent, and the procedures were approved by the Institutional Review Board of the University of California at Los Angeles, and comply with the Declaration of Helsinki.

### Data Acquisition

Data were acquired with a General Electric 1.5 T Magnetic Resonance Imaging unit. Participants lay supine and breathed through a mouthpiece connected to a two-way non-rebreather valve. End-tidal CO_2_ and ECG signals were measured and transmitted to a recording system outside the scanner room. These signals were subsequently used to calculate heart and respiratory rates. For monitoring purposes, pulse oxygen saturation was also measured. The fMRI data consisted of gradient-echo echo-planar images (repetition time [TR] = 6,000 ms per volume, time to echo [TE] = 60 ms, flip angle = 90°, field of view [FOV] = 30 × 30 cm, no interslice gap, voxel size = 2.3 × 2.3 × 5.0 mm) composed of 20 oblique sections, and the protocol was a 144 s series of 24 volumes with gas challenges administered 24 s into the series and lasting for the remaining 120 s. Series were collected during each of three conditions: baseline (no gas administered), hyperoxia (100% O_2_), and hypercapnic hyperoxia (5% CO_2_, balance O_2_). Gas mixtures were delivered via the inspiratory arm of the valve. A minimum of 8 min elapsed between challenges, allowing the participants to return to a baseline state, as verified by physiologic signals.

### Statistical Analysis

Data were analyzed using the Statistical Parametric Mapping software SPM (http://www.fil.ion.ucl.ac.uk/spm). Each fMRI series was corrected for timing differences in slice acquisition, and motion-corrected; series with more than 4 mm of translation or more than 1° of rotation in any direction were excluded from the analysis. Images were then spatially normalized (full details are described elsewhere [21]) and segmented into gray matter, white matter, and CSF. The segmentation relies upon signal intensity differences between gray and white matter regions in the fMRI images, and atlas-based probability distribution maps of the tissue types [22]. The technique is robust to distortions in the images; partial volume effects arise due to the low resolution of the fMRI images, but a probability threshold of 0.5 for classification of gray matter ensured that the majority of gray matter regions were included in gray matter images. The fMRI images are sensitive to changes in blood-oxygen levels; the signal is derived from the relative proportion of deoxygenated and oxygenated blood (termed blood-oxygen level dependent [BOLD] signal). Hyperoxia and hypercapnia cause global changes in the BOLD signal from CBF and oxygenation alterations, which confound the effect of interest (local changes in blood oxygenation—and hence in the BOLD signal—corresponding indirectly to changes in local field potentials and hence neuronal activation). The magnitude and timing of the global signal changes differ between white and gray matter [23,24]; thus, only the latter images were analyzed. Within each individual's gray matter fMRI time series, all global effects were removed using a conservative detrending method [25]. The detrended images were smoothed (full-width-half-maximum 8 mm).

The two gas challenges were analyzed separately. A linear model was constructed with five step-function independent variables for each child, set to 1 (“on”) during each of five 24-s periods of the challenge and 0 (“off”) otherwise. The model was convolved with the SPM standard hemodynamic response function. The baseline series was included during the “off” period. Each series also included a constant term to account for variations in absolute signal intensity. The signal intensity at each voxel (i.e., each “pixel” or sample point in the image volume) across individuals was modeled as a linear function of the step-function variables and constants, and tested for positive or negative effects (*F*-test), corresponding to increasing or decreasing signal intensity during the challenge (termed a fixed-effects analysis). The *F*-test findings indicated absolute responses, regardless of the direction of signal changes.

All analyses were performed across the group of 14 participants. Each voxel was evaluated using a significance threshold of *p* < 0.05, with false discovery rate correction for multiple comparisons [26]. The results from all voxels were combined into a whole-brain statistical “map” of areas showing significant signal changes. As each time-period was evaluated independently, a map was generated for each 24 s period of a gas challenge. Statistical effect sizes were calculated using Pearson's *r* calculated from the formula

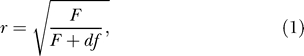
where *F* is the *F* ratio and *df* degrees of freedom [27]. The maximum and minimum effect sizes for regions in each significance map were presented, as opposed to whole-brain effect size maps. Each significance map was presented as a colored overlay onto a background high-resolution anatomic image. In addition, a summary presentation combining all time points of significant response was created for the two gas challenges, providing an overview of all responsive regions.

These data were previously used in part as control for examination of responses to chemoreceptor challenges in patients with congenital central hypoventilation syndrome (CCHS) [28-30]. In these earlier studies, hyperoxia responses in the normal participants were not examined separately, and a different analysis (population-level “random-effects” analysis, single boxcar) was used to evaluate the normal responses to hypercapnia. These data were also used to study global BOLD signal responses in the patient group [23], and to devise a method to remove such effects [25]. Physiologic data were analyzed previously as part of an investigation into CCHS and have been reported separately [31].

## Results

The physiologic patterns of response to the challenges, reported earlier in a subset of 12 of the current 14 children [31], were similar to those reported elsewhere. The expected decline in heart rate due to hyperoxia [9] appeared after one minute. Respiratory rate showed a brief increase; tidal volume, which was not measured, is responsible for the majority of the reported increased ventilation due to hyperoxia, with respiratory rate not greatly affected [32,33]. Hypercapnia induced a sustained rise in both respiratory and cardiac rates, as anticipated [34]. Trends in end-tidal CO_2_, an increase due to hypercapnia and decrease to hyperoxia, were also consistent with the established physiologic literature.

**Figure 1:**
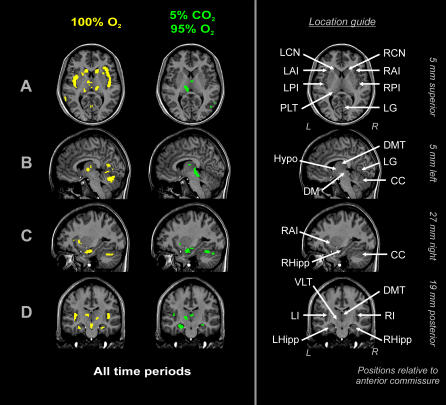
Summary of Regions of Significant Response to Gas Challenges Yellow (hyperoxia) and green (hypercapnic hyperoxia) overlays of regions of significant signal response in 14 children at any of five time successive periods during the challenges. Multiple brain regions responded to hyperoxia (100% O_2_), but the addition of 5% CO_2_ to the gas mixture greatly reduced most responses. Statistical threshold *p* = 0.05, false discovery rate correction. Location guide key: CC, cerebellum; DMT, dorsal medial thalamus; Hypo, hypothalamus; LAI/RAI, left/right anterior insula; LAP/RAP, left/right posterior insula; LCN/RCN, left/right caudate nucleus; LG, lingual gyrus; LHipp/RHipp, left/right hippocampus; LI/RI, left/right insula; PLT, posterior lateral thalamus; VLT, ventral lateral thalamus. Distances from the anterior commissure and orientation, based on the standard Montreal Neurological Institute space, are (A) 5 mm superior, (B) 5 mm left, (C) 27 mm right, and (D) 19 mm posterior. Distance increases from left-to-right for sagittal (side) views, posterior-to-anterior for coronal views, and inferior to superior for axial (transverse) views. The background image is a high-resolution scan from a single participant (normalized to Montreal Neurological Institute space).

Hyperoxia elicited a sequence of fMRI responses in the posterior hypothalamus, insula, hippocampus, cerebellum, caudate, and thalamic regions (Figure 1). The areas highlighted in Figure 1 exhibited signal patterns that increased or decreased during any of the successive time periods. The addition of 5% CO_2_ to the hyperoxic mixture greatly reduced or eliminated responses in most structures (Figure 1). The hypercapnic hyperoxia challenge elicited responses in discrete additional areas, in particular, the dorsal midbrain. Effect sizes in regions of significant response ranged from small (0.1) to medium (0.3; Table 1).

**Table 1:**
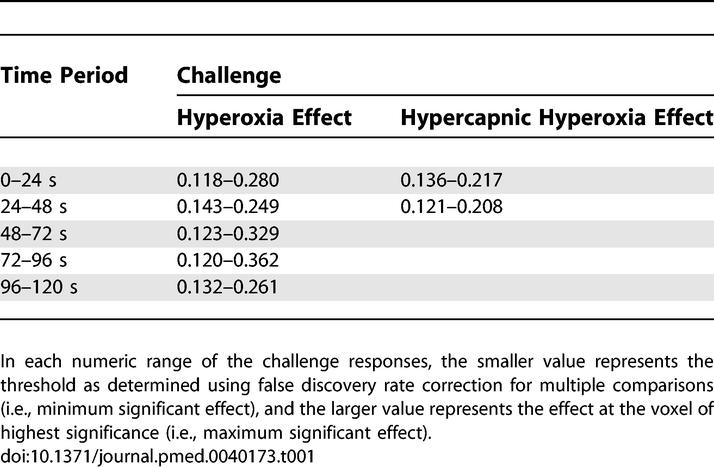
Overview of Effect Sizes Calculated Using Pearson's *r* for Regions of Significant Response

**Figure 2:**
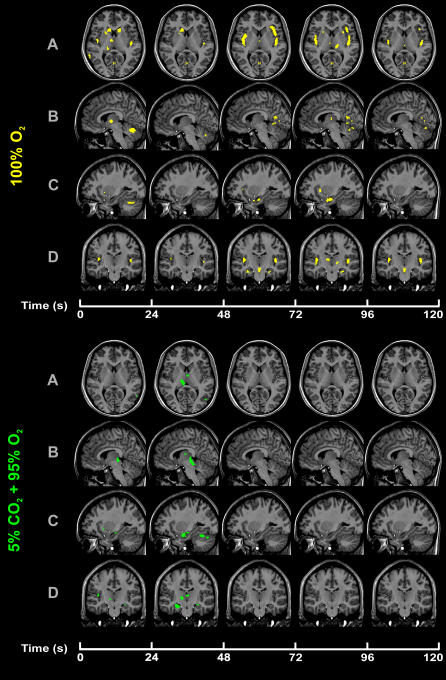
Time-Specific Regions of Response to Gas Challenges Hyperoxia (top rows, yellow) and hypercapnic hyperoxia (bottom rows, green) overlays of regions of significant signal response in 14 children at five successive time periods during the challenges (time scale below images). Location guide and views (A–D) are the same as in Figure 1. As shown by images in the top rows, multiple brain regions responded to hyperoxia, but addition of 5% CO_2_ to the gas mixture altered the timing or greatly reduced most responses (bottom rows), and eliminated all responses after 48 s. Statistical threshold *p* = 0.05, false discovery rate correction.

The responses varied across time, as shown in Figure 2. Some structures, such as the caudate and hypothalamus, responded to hyperoxia during the initial 24–48 s, whereas other structures, such as the insula and cerebellum, responded throughout the challenge. Most notably, compared to 100% O_2_, hypercapnic hyperoxia resulted in fewer regions of immediate response, and eliminated all responses after the first 48 s of the challenge.

The insula responded to hyperoxia alone bilaterally in posterior regions during the first 24 s, followed by a reduction in response up to 48 s. Subsequently, the entire structure was recruited, with a greater extent of the anterior right insula involved. The addition of CO_2_ reduced the insula response to only the first 24 s, and with smaller regions of response occurring more anteriorly than the initial hyperoxic changes.

The 100% O_2_ challenge elicited hypothalamic and posterior thalamic changes within the first 24 s, following which reduced responses appeared in thalamic regions for the remainder of the challenge. The hypothalamic responses to hyperoxia disappeared with addition of CO_2_, as did bilateral signal changes in the head of the caudate. The head of the caudate showed bilateral early responses to 100% O_2_, but only left-side reactions after 24 s. The hypercapnia/hyperoxia challenge elicited signal changes in the posterior and mid-thalamus similar to those found with hyperoxia, showing the greatest responses in the 24–48 s period.

Signal changes in the dorsal hippocampus to 100% O_2_ appeared late, after 48 s, and to a greater extent on the right side. The addition of 5% CO_2_ elicited immediate responses in the bilateral ventral hippocampi, rather than the dorsal portions; this pattern was sustained during the first 48 s of the challenge.

The cerebellum showed immediate and extensive responses to hyperoxia, with both cerebellar cortex and deep nuclei patterns developing during the first 48 s. The addition of CO_2_ eliminated most cerebellar changes, with muted reactions appearing only during the 24–48 s period of the challenge, and only in the cerebellar cortex.

Isolated regions of response to hyperoxia appeared in the medial lingual gyrus of the occipital lobe throughout the challenge, but disappeared with the addition of CO_2_. Additional small, isolated regions in the posterior ventral–lateral parietal cortex emerged with both challenges within the first 48 s.

## Discussion

We found that a number of brain areas responded to a hyperoxic challenge, especially neural regions that mediate autonomic and hormonal systems. However, addition of 5% CO_2_ to the hyperoxic mixture substantially reduced reactions of these neural structures. The perfusion and hormonal changes to 100% O_2_ could initiate a cascade of central and peripheral injuries through oxidative stress processes commonly reported with high oxygen ventilation. Since the structures recruited in hyperoxia control output of hypothalamic sympathetic and hormonal regulatory areas, the reduced responses of those structures with the addition of CO_2_ may diminish injury to central and peripheral organs following hyperoxia alone, a possibility suggested by others [6].

The brain regions activated in hyperoxia have long been known to project heavily to hypothalamic areas mediating autonomic and hormonal regulation. The insular cortex projects to hypothalamic and amygdala sites, and recording, stimulation, and stroke injury evidence demonstrates a lateralized and topographic organization of parasympathetic, sympathetic, and baroreflex regulation for that structure in animals and humans [16–18,35–39]. The substantial recruitment of the insula in hyperoxia, especially on the right side, likely reflects a role in mediating sympathetic components of the challenge. In the absence of CO_2_, the insula remains recruited throughout the challenge period, consistent with a sustained increase in sympathetic outflow; abolition of this response with addition of CO_2_ undoubtedly modifies influences on hypothalamic sites, decreasing sympathetic tone.

Cerebellar cortex and deep nuclei apparently serve a role in modifying blood pressure and in limiting extremes of blood pressure change [37,40,41], including recruitment of motor actions to compensate for hypotension [42], and may serve a similar role for sympathetic action during hyperoxia. Cerebellar cortex and deep nuclei were extensively recruited during the first 24 s of the hyperoxia challenge, corresponding to the initial period of vasoconstriction and the accompanying blood pressure increase. Reduced hypothalamic sympathetic outflow via insular responses, combined with CO_2_ counteracting O_2_-induced vasoconstriction, would result in more modest blood pressure changes, and hence a reduced need for recruitment of the cerebellum to modulate the changes; indeed, cerebellar responses to CO_2_ addition were reduced or eliminated from those of hyperoxia alone.

The hippocampus has pronounced anatomic projections to the hypothalamus as well as to the insula and amygdala [35,43,44], reacts to electrical stimulation with blood pressure increases [45], and activates with blood pressure manipulation [14] or respiratory patterns that elicit blood pressure changes [46]. The dorsal hippocampus was recruited later in the hyperoxia challenge. The addition of CO_2_ resulted in larger initial ventral hippocampal responses, especially from 24 to 48 s, with later changes abolished. The precise function of the hippocampal response to these challenges remains speculative, but may be related to blood pressure interactions with respiratory changes; the respiratory changes occur early in response to CO_2_, but over a longer period of time to 100% O_2_.

An area showing immediate recruitment in response only to the hypercapnic/hyperoxic mixture, but not to hyperoxia, was the dorsal midbrain, with more extensive responses during the 24–48 s challenge period. The responsive region included dorsal portions of the periaqueductal gray matter, which contains respiratory neurons with poorly known influences on respiratory patterning in the cat [47]; stimulation of the dorsal periaqueductal gray region also elicits effects on respiratory musculature [48,49]. It is unclear whether a portion of the reduction of hypothalamic and insular signals during hypercapnic hyperoxia results from processes induced by dorsal midline recruitment.

The findings suggest that the common medical practice of hyperoxic gas administration may benefit from CO_2_ supplementation through reduction of central neural responses accompanying hyperoxia-induced sympathetic outflow and hormonal release. A portion of the often detrimental peripheral outcomes, e.g., alterations in myocardial contractility, reduced myocardial relaxation, and certain hormonal effects such as increased glucagon levels, may result from alterations in autonomic site function in the brain.

Supplementation of hyperoxia with CO_2_ does reduce induction of hypocapnia and consequent reductions in CBF, and alleviates damage due to oxygen stress. The biochemical processes associated with tissue injury in oxygen stress have been well-outlined [50,51], as have interventions to prevent such injury [52]. CO_2_ supplementation for neonatal resuscitation is indicated by suggestions that room air may be at least as effective as 100% O_2_ in infants subjected to asphyxia [53,54] and that biochemical correlates of oxygen stress are diminished [55]. Room air resuscitation results in less injury to the heart and kidney [56]; injury to the heart is especially apparent after hyperoxic exposure [57]. Similarly, hyperbaric O_2_ fared no better than pressurized air for improvement in gross motor function when administered to patients with cerebral palsy [58], and hyperoxia and hypocapnia add to the risk of brain injury after intrapartum asphyxia [59]. Hyperoxic ventilation is often administered for treatment of stroke; however, the accompanying hypocapnia and resultant CBF reductions and diminished O_2_ delivery may be contraindicated, as for resuscitation. The perinatal ischemic rodent brain is better protected with addition of mild hypercapnia to the ventilatory supplement mix [60].

It should be noted that patients requiring resuscitation often show initially high CO_2_ levels following respiratory failure, and an argument could be made that under such circumstances the addition of CO_2_ to hyperoxic delivery is unnecessary. However, CO_2_ levels rapidly dissipate with forced ventilation, and hypocapnia would ensue after a short period, leading to the sequence of constricted vasculature described earlier.

It is important to note that children were used in the present study. The findings may not extend to adults, or to neonates, in whom resuscitation with hyperoxia is much more of a concern. We expect that the findings will generalize at least to adults, as a previous fMRI study of a hyperoxic hypercapnic challenge in a mixed adult/pediatric population shows comparable results in the dorsal pons and cerebellum [61]; the previous study was limited to midline areas.

It should be cautioned that determination of local activity within a regional brain area with fMRI depends on deoxygenation/oxygenation changes within those regions. Such measures are confounded by global changes in blood flow and deoxyhemoglobin levels associated with the gas challenges. Although the gas challenges induce major changes in CBF, the detrending method used ensures that regional responses found in the fMRI data are unlikely to result from changes in CBF [25]. CBF changes occur throughout the vasculature, and therefore are represented in the data as a change in the global signal, i.e., the average intensity signal across the whole brain. We developed a detrending method that removes any component of the data matching the global signal [25]; for any remaining signal to correspond to changes in CBF, those changes would have to be of a different timing and amplitude pattern than the majority of the CBF changes, which is unlikely given the interconnected nature of the cerebral vasculature. Minor timing differences in CBF response between regions will not affect the detrending as the fMRI volumes were collected at six-second intervals. We also segmented gray and white matter, as the vascular response differs slightly between these two tissue types [23,24]. Given the segmentation of gray matter and the application of conservative detrending, the findings should represent primarily regional responses.

One consequence of the detrending method used is that any regional responses corresponding to neural activity changes that follow a similar time course to the vascular changes are removed. Therefore, the results likely do not represent all of the neural activation–related signal changes that occurred with the challenges.

A final consideration is the potential influence of the varying CO_2_ and O_2_ levels on the magnitude of the BOLD effect; these gases can alter the extent of neurally related hemodynamic changes [62,63]. However, although the BOLD effect is influenced by extreme changes in baseline CO_2_ (e.g., 10%) and O_2_ (e.g., 9%), the regional effect is robust across a range of hypercapnic, hypoxic, and hyperoxic conditions. Measurements in rats show that the regional hemodynamic response is similar under 5% CO_2_ hypercapnia to normocapnic conditions [62], with comparable findings in humans with 4% CO_2_ [64]. Increases (up to 100% O_2_) and moderate decreases in oxygenation also do not lead to changes in regional BOLD responses [63]. With the current study's 5% CO_2_ and hyperoxic conditions, if changes in the BOLD effect did occur, they likely had little influence on the findings.

In summary, hyperoxic ventilation modified activity in several rostral brain structures that regulate autonomic and hormonal outflow, and these modifications may underlie a portion of the adverse autonomic and hormonal changes associated with treatment. Addition of CO_2_ to the hyperoxic gas mixture reduced the neural responses found during hyperoxia alone, and the response decline in neural modulatory areas may serve to mitigate the deleterious physiologic consequences of high oxygen.

## Abbreviations

BOLD: blood-oxygen level dependent
CBF: cerebral blood flow
fMRI: functional magnetic resonance imaging
